# NONHSAT141192.2 Facilitates the Stemness and Radioresistance of Glioma Stem Cells via the Regulation of PIK3R3 and SOX2

**DOI:** 10.1111/cns.70279

**Published:** 2025-02-19

**Authors:** Sihan Wang, Haolang Ming, Zhen Wang, Xingye Zhai, Xinyue Zhang, Di Wu, Yin Bo, Hang Wang, Yuanbo Luo, Zhenfeng Han, Lingyu Hao, Yijia Xiang, Xu Han, Zengguang Wang, Yi Wang

**Affiliations:** ^1^ Department of Neurosurgery Tianjin Medical University General Hospital Tianjin China; ^2^ Tianjin Neurological Institute, Key Laboratory of Post‐Trauma Neuro‐Repair and Regeneration in Central Nervous System, Ministry of Education & Key Laboratory of Injuries Variations and Regeneration of Nervous System in Tianjin Tianjin China

**Keywords:** glioblastoma multiforme, glioma stem cells, long non‐coding RNA, Radioresistance

## Abstract

**Background:**

Glioma stem cells (GSCs) contribute to the initiation, recurrence, metastasis, and drug resistance of glioblastoma multiforme (GBM). Long noncoding RNAs (lncRNAs) are critical modulators in the development and progression of GBM; however, specific lncRNAs related to GSCs remain largely unexplored. This study aims to identify dysregulated lncRNAs in GSCs, unravel their contributions to GBM progression, and propose new targets for diagnosis and treatment.

**Methods:**

GeneChip analysis was utilized to identify lncRNAs in GSCs. The expression of RNAs was examined using quantitative real‐time PCR. Cell Counting Kit‐8, tmorsphere formation assay, limiting dilution assay, apoptosis detection and intracranial xenograft models were performed to assess the stemness and radioresistance of GSCs. Transcriptomics analysis, RNA immunoprecipitation and dual‐luciferase experiments were conducted for mechanistic studies.

**Results:**

NONHSAT141192.2 exhibited elevated expression levels in aggressive GBM tissues compared to lower‐grade gliomas. Silencing NONHSAT141192.2 resulted in a considerable decrease in GSC proliferation, tumor sphere formation, self‐renewal and the expression of key stem cell markers. Furthermore, depletion of NONHSAT141192.2 enhanced GSC sensitivity to radiation, indicated by diminished viability and tumorsphere formation, increased cell apoptosis, and decreased tumor growth in intracranial xenograft models. Mechanistically, NONHSAT141192.2 upregulates the expression of SOX2 and PIK3R3 by sponging miR‐4279, influencing GSC characteristics and their resistance to radiation.

**Conclusion:**

The study highlights a significant relationship between NONHSAT141192.2, GSC stemness, and radioresistance, emphasizing its potential as a therapeutic target for GBM treatment and radiosensitization.

## Graphical abstract

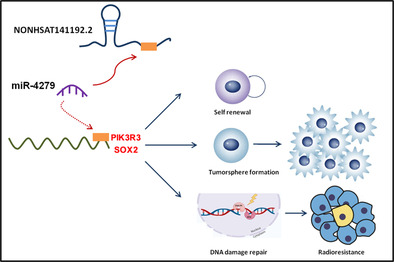

## 1 Introduction

Glioblastoma multiforme (GBM) is the prevalent and highly aggressive type of brain cancer, constituting nearly 50% of malignant brain tumors in adults [1]. The standard treatments for GBM typically involve surgical therapy combined with concurrent chemoradiotherapy [2, 3]. However, due to its aggressive nature, complete surgical removal of GBM is often difficult, leading to high recurrence rates and poor patient prognosis. Despite advancements in adjuvant therapies such as chemoradiotherapy and targeted treatments, the recurrence rate remains alarmingly high. The average survival time for patients with GBM is only eight months, with a mere 6.9% of patients surviving five years post‐diagnosis [4]. Within GBM, a subpopulation known as glioma stem cells (GSCs) exhibits significant self‐renewal, tumor‐initiating, and tumor‐maintaining capabilities. GSCs are characterized by their invasive growth and resilience to both radiation and chemotherapy, making them a critical factor in glioma recurrence and treatment resistance [5, 6]. Understanding the mechanisms that sustain the features of GSCs may provide valuable insights for developing more effective GBM treatments.

Long noncoding RNAs (lncRNAs) refer to a class of RNA molecules that lack coding capacity and consist of over 200 nucleotides [7]. Recent studies have emphasized the regulatory roles of lncRNAs in various biological processes, including transcription, translation, and posttranslational modifications [8, 9, 10]. These molecules have been implicated in various cellular functions such as proliferation, invasion, metabolic regulation, therapeutic resistance, and maintaining stem cell properties [11, 12, 13]. Previous research has revealed that many lncRNAs exhibit dysregulated expression in GBM. For example, lncRNA LUCAT1 enhances GSC self‐renewal and GBM progression by augmenting hypoxia‐inducible factor 1 alpha activity [14]. Additionally, lncRNA INHEG exhibits higher expression levels in GSCs compared to differentiated glioma cells, promoting GSC self‐renewal and tumorigenicity through the regulation of rRNA 2’‐O‐methylation [15]. Furthermore, TGF‐β‐activated lncRNA LINC00115 serves as a critical regulator of GSC self‐renewal and tumorigenicity [16]. These findings suggest a potential role of lncRNAs in the molecular mechanisms driving GBM pathogenesis. However, only a limited number of lncRNAs in determining the functional characteristics of GSCs have been functionally characterized to date.

In this study, we identify a significantly upregulated lncRNA, NONHSAT141192.2, in GSCs, which elevates the expression of sex‐determining region Y‐box 2 (SOX2) and phosphoinositide‐3‐kinase regulatory subunit 3 (PIK3R3), by sponging miR‐4279. This process supports GSC self‐renewal and mitigates DNA damage, ultimately contributing to radioresistance. Overall, our study offering a further understanding of NONHSAT141192.2 in GSCs and GBM, suggesting it as a potential therapeutic target for GBM treatment.

## 2 Methods

### 2.1 Tumor Specimens

In total, we collected 17 fresh glioma samples from patients diagnosed with GBM and lower‐grade gliomas (grades I–III) at Tianjin Medical University General Hospital between June 2022 and September 2023. The patient inclusion criteria included adults aged 18 years and older who were newly diagnosed with glioma and had not received any prior treatment. Exclusion criteria included patients with secondary gliomas or those who had undergone any preoperative therapies. The clinical and pathological characteristics of these patients are summarized in Table 1. Each glioma specimen was histologically confirmed by two qualified pathologists according to the World Health Organization grading system. All procedures involving human samples were conducted in accordance with the principles outlined in the Helsinki Declaration and were approved by the Ethics Committee of Tianjin Medical University General Hospital (Approval No: IRB2022‐KY‐234; Date: 20220310). Written informed consent was obtained from all participants.

**TABLE 1:** Information of samples used in this study.

Characteristic	Number (*n* = 17)
Age (years)	52.65 ± 18.11
Gender, Male/Female	9/8
Grade, I/II/III/IV	1/2/1/13
KPS score, < 80/≥ 80	9/8
Location, Left/Right	10/7
Size, < 3.5 cm/ ≥ 3.5 cm	7/10
Peritumoral edema, Mild/Serious	5/12

### 2.2 Cell Culture

LN229 cells (CL‐0578, Procell, Shanghai, China) were cultured in Dulbecco's modified Eagle's medium (DMEM) with 5% fetal bovine serum (FBS). U251 cells (CL‐0237, Procell, Shanghai, China) were cultured in DMEM supplemented with 10% FBS in a humidified incubator at 37°C with 5% CO_2_.

The LN229‐GSC and U251‐GSC, the undifferentiated forms of LN229 cells and U251 cells, were maintained in a GSC‐induced medium containing 4 μg/mL insulin, B27 (1:50, Invitrogen, USA), 20 ng/mL epidermal growth factor (EGF; R&D, USA) and 10 ng/mL basic fibroblast growth factor (bFGF; R&D, USA) in 10 cm low‐attachment plates (Corning, USA).

### 2.3 Patient‐Derived GSC Generation and Cell Culture

Patient‐derived GSCs were generated from the surgical specimens of primary GBM. In brief, tumor specimens were cut into small pieces with a sterile scissor and digested in an enzyme solution mixed with collagenase IV (Gibco, Vacaville, CA, USA) and DNase1 (Sigma, Burlington, MA, USA) at 37°C for 2 h. The obtained GSCs were cultured in serum‐free DMEM/F12 medium containing 20 ng/mL human recombinant EGF, 10 ng/mL FGF‐2, and 1× GlutaMax (Invitrogen, USA). GSC03 was derived from a female patient with GBM.

### 2.4 RNA Extraction

Total RNA was extracted from U251‐GSC, LN229‐GSC, U251, and LN229 cells using the RNeasy Plus Mini Kit (Qiagen, Valencia, CA) following the manufacturer's instructions. RNA quality and integrity were assessed using NanoDrop microspectrophotometer (Thermo Scientific) and Qubit 3.0 Fluorometer (Agilent Technologies). Only samples with an RNA integrity number (RIN) greater than 8.0 were used for subsequent analyses.

### 2.5 GeneChip Whole Transcriptome Analysis

Transcriptome analysis was performed using the GeneChip Whole Transcript Expression Array (Affymetrix, Santa Clara, CA). Labeling of the RNA was performed using the GeneChip WT Pico Reagent Kit (Affymetrix), following the manufacturer's guidelines for first‐strand cDNA synthesis, amplification, and biotin labeling. The cDNA was then fragmented and hybridized to the GeneChip WT Pico Reagent Kit arrays, which provide comprehensive coverage of human transcripts, including protein‐coding genes and non‐coding RNAs.

After hybridization, arrays were washed and stained using the GeneChip Fluidics Station 450 (Affymetrix) according to standard protocols. Scanning of the arrays was performed with the GeneChip Scanner 3000 7G (Affymetrix).

### 2.6 Data Processing and Analysis

Raw data from the GeneChip arrays were processed using the Affymetrix Expression Console software. Probe set normalization and background correction were performed using the RMA (Robust Multi‐array Average) method. Differential gene expression analysis was conducted using the limma package in R (version 4.0.2) to compare the transcriptomes of U251‐GSC and LN229‐GSC cells with their respective parental lines (U251 and LN229). A threshold of |log2 fold change| > 1 and an adjusted p‐value < 0.05 were used to identify differentially expressed genes.

Gene Ontology (GO) and pathway enrichment analysis were performed using the DAVID (Database for Annotation, Visualization and Integrated Discovery) Bioinformatics Resource and Kyoto Encyclopedia of Genes and Genomes (KEGG) pathways.

### 2.7 Establishment of Stable Expression Cell Lines

Lentivirus‐packaged sh‐NONHSAT141192.2 (sh‐lncRNA) and control (sh‐NC) were transfected into GSCs. The detailed shRNA sequences are listed in Table 1. For SOX2 or PIK3R3 overexpression analysis, lentivirus‐packaged SOX2 (SOX2_OE) or PIK3R3 (PIK3R3_OE) and control (vector) were transfected into GSCs. These lentiviruses, miR‐4279 mimics, and inhibitors were provided by GenePharma (Shanghai, China). To obtain stably transfected cells, the infected cells were cultured in a medium containing 1 μg/mL puromycin (Gibco, USA) for 2 weeks.

### 2.8 Quantitative Real‐Time PCR (QRT‐PCR)

The isolation of Total RNA was performed using a TRIzol reagent (Beyotime, China). PrimeScript RT reagent Kit (TaKaRa, Japan) was utilized for cDNA synthesis. Real‐time PCR was conducted using SYBR Premix ExTaq II (TaKaRa, Japan). The 2^‐ΔΔCT^ method was applied to calculate relative expression levels. The primer sequences are listed in Table 2.

**TABLE 2:** The sequence of primers and shRNAs.

Genes	Sequences
NONHSAT051892	5’ CCCGAGACACCACGCAC 3′ 5’ GTGTCACGGGACGGGTG 3’
NONHSAT141192.2	5’ TGAGATGGGAGGATTGCTT 3′ 5’ TGTTGTTATTGGAGACAGGGT 3’
NONHSAT1410245	5’ ACCGATGGGAATTTAGGTGA 3′ 5’ GGTGTTGGTGTTGTCGCAGTA 3’
hsa‐miR‐4279	RT: GTCGTATCCAGTGCAGGGTCCGAGGTGCACTGGATACGAC GAAGCCG FWD: TGCGG GAGACGAGGGCCGAAG Reverse: CCAGTGCAGGGTCCGAGGT
hsa‐miR‐372‐5p	RT: GTCGTATCCAGTGCAGGGTCCGAGGTGCACTGGATACGAC AGAATAG FWD: TGCGG GGAGTTTACACCTCGTG
hsa‐miR‐578	RT: GTCGTATCCAGTGCAGGGTCCGAGGTGCACTGGATACGAC ACAATCCT FWD: TGCGG GAAGAACACGAGATCCTAA
SOX2	5’ GTGGTTACCTCTTCCTCCCACTCCAGG 3′ 5’ TGTGCCGTTAATGGCCGTGCC 3’
miR‐4279 mimics	5’ GAAGCCGGGAGGAGAG TT 3’
NONHSAT141192.2	ShRNA‐1: 5’ GAGAAGGTGACAATCGAAATGAAAT 3’ ShRNA‐2: 5’ GGTGACAATCGAAATGAAATGCTTT 3’

### 2.9 Western Blotting Analysis

The following primary antibodies were used in this study: SOX2 (1:500, ab97959, Abcam), γ‐H2AX (1:500, ab26350, Abcam), ATM (1:500, ab17995, Abcam), p‐ATM (Ser1981) (1:500, ab308338, Abcam), NESTIN (1:500, 66259‐1‐Ig, Proteintech), OCT‐4 (1:500, 60242‐1‐Ig, Proteintech), α‐Tubulin (1:5000, 11224‐1‐AP, Proteintech), GAPDH (1:500, 60004‐1‐Ig, Proteintech).

### 2.10 Cell Viability Assays

Cell viability was measured using the Cell Counting Kit‐8 (CCK‐8) assay kit (Beyotime, China). GSCs were seeded in 96‐well low‐attachment plates (1 × 10^3^ cells per well). Following incubation for 24, 48, and 72 h, 10 μL of CCK‐8 was added to each cell. After 2 h of incubation, the cells were measured at 450 nm by a microplate reader.

### 2.11 Tumorsphere Formation Assay

U251‐GSC or GSC03 cells (5 × 10^2^ cells/well) were plated in 96‐well low‐attachment plates (Corning, USA) and maintained in the GSC medium. Following a period of 10–14 days, tumorspheres were examined using a microscope, and the number and diameter were calculated.

### 2.12 Limiting Dilution Assay

Treated U251‐GSC or GSC03 cells were inoculated into 96‐well low‐attachment plates by 100, 50, 10, and 1 cells/well, and 50 wells were inoculated at each concentration. The cells were cultured in a serum‐free stem cell medium in a humidified incubator at 37°C with 5% CO_2_. After 14 days, the number of wells without sphere formation was counted. Linear discriminant analysis (LDA) software (https://bioinf.wehi.edu.au/software/elda/) was used to analyze the frequency of stem cells.

### 2.13 Immunofluorescence Staining

U251‐GSC or GSC03 cells (5 × 10^4^ cells/well) were spread on glass coverslips in a 24‐well plate and cultured overnight. After 24 h, the cells were fixed in 4% paraformaldehyde for 15 min and then incubated in PBS containing 0.1 M glycine for 5 min to terminate the fixation reaction. The cell coverslips were placed on the sealing film and sealed with 100 μL of sealing liquid at room temperature for 40 min. After removing the sealing liquid, the cells were incubated with 100 μL of primary antibody diluent at room temperature for 4 h. This was followed by incubation with a secondary antibody at room temperature for 2 h. Then, the cells were stained with diluted DAPI (1:400) for 3 min. After being sealed with 4 μL of anti‐fluorescence quenching sealing solution, the cells were photographed and analyzed under a fluorescence microscope.

### 2.14 Intracranial Mouse Model

Nude mice aged 4–6 weeks were obtained from Vital River for this study. luciferase GSCs were transduced with lentiviral vectors expressing NONHSAT141192.2 shRNA (sh‐lncRNA) or NC shRNA (sh‐NC) for the knockdown experiments. A total of 3 × 10^5^ viable GSC03 cells were stereotactically implanted into the brains of the mice (12 mice for each group). Starting on day 3 post‐implantation and continuing until day 7, half of the mice received daily whole‐brain irradiation at a dose of 2 Gy. Bioluminescence imaging was performed to monitor intracranial tumor growth on days 14, 21, and 28. Mice were observed until they exhibited neurological symptoms, at which point they were euthanized. Kaplan–Meier survival curves were constructed to assess overall survival. Tumor tissues were harvested and fixed in 4% paraformaldehyde. Following fixation, the tissues were embedded in paraffin and sectioned to a thickness of 4 μm for immunohistochemical analysis. All procedures involving animals complied with the statement for the Use of Animals in the Basel Declaration, and ethical approval was granted by the ethics committee of Tianjin Medical University General Hospital (Approval No: IRB2022‐DWFL‐329; Date: 20220310).

### 2.15 Statistical Analysis

Data are presented as the means ± standard deviation (SD). Statistical analysis was performed using SPSS 23.0. For quantitative data, Student's t‐test was utilized for comparisons between two groups, while one‐way or two‐way analysis of variance (ANOVA) was applied for comparisons involving three or more groups. The Shapiro–Wilk test was applied to assess the normality of experimental data distributions. Fisher's Exact Test was performed to assess the association between NONHSAT141192.2 expression and clinicopathological factors. A p‐value < 0.05 was considered to be statistically significant.

## 3 Results

### 3.1 Differently Expressed lncRNAs Between GSCs and Differentiated GSCs

**FIGURE 1:**
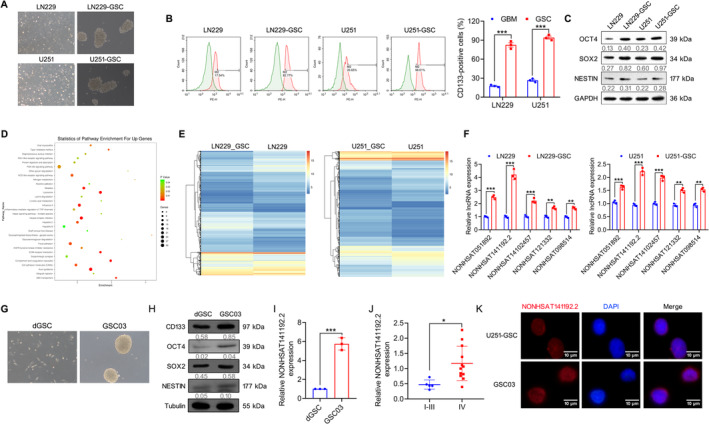
The identification and characteristics of NONHSAT141192.2 in GBM. (A) Induction of GSCs from LN229 and U215 glioma cells using GSC‐induced medium. (B) Flow cytometry analysis of stem cell marker CD133 (*n* = 3). (C) Western blotting analysis of stem cell markers including OCT4, SOX2, and NESTIN in GBM cells and GSCs (*n* = 3). (D) KEGG enrichment of upregulated differentially expression genes between GSC and non‐GSC cells. (E) Heatmaps of lncRNAs that were differentially expressed between GSC and non‐GSC cells. (F) Expression of the top five lncRNAs in GSCs and non‐GSCs was measured by qRT‐PCR (*n* = 3). (G) Isolation of patient‐derived GSC03 cells. (H) Western blotting analysis of stem cell markers including CD133, OCT4, SOX2, and NESTIN in GSC03 and dGSC (*n* = 3). (I) The expression of NONHSAT141192.2 in patient‐derived GSC03 and dGSC was measured by qRT‐PCR (*n* = 3). (J) The expression of NONHSAT141192.2 in I‐III glioma (*n* = 4) and GBM (*n* = 13) tissues. (K) FISH assay was performed to measure the localization of NONHSAT141192.2 in U251‐GSC and GSC03. Scale bar = 10 μ0. *p < 0.05, **p < 0.01, ***p < 0.001.

Human GBM cell lines U251 and LN229 were induced into glioma stem‐like cells, named U251‐GSC and LN229‐GSC, and showed a typical tumorsphere‐like growth pattern (Figure 1A). Flow cytometry analysis revealed that the positive rate of CD133, a representative stem cell marker, in U251‐GSC and LN229‐GSC was significantly increased, even reaching 98% (Figure 1B). Furthermore, western blotting analysis demonstrated that the expression levels of octamer‐binding protein 4 (OCT4), SOX2, and Nestin were significantly higher in U251‐GSC and LN229‐GSC cells than those in their parental cells (Figure 1C). These data confirmed the stem‐like characteristics of U251‐GSC and LN229‐GSC cells.

Next, we performed transcriptome analysis of U251‐GSC and LN229‐GSC cells relative to their parental cells using GeneChip Whole Transcript Expression Arrays. Kyoto Encyclopedia of Genes and Genomes (KEGG) enrichment analysis indicated that the upregulated differentially expressed genes (DEGs) in GSCs were enriched in retinoic acid‐inducible gene I (RIG‐I)‐like receptors, phosphatidylinositol 3‐kinase (PI3K)‐Akt signaling pathway, and Hippo signaling pathway (Figure 1D), which are related to the growth and self‐renewal of cancer stem cells [17, 18, 19]. Notably, 670 lncRNAs were identified that were differentially expressed in both U251‐GSC and LN229‐GSC cells compared with their parental cells (false discovery rate < 0.01, and fold change > 2), including 359 upregulated and 194 downregulated lncRNAs in both U251‐GSC and LN229‐GSC cells (Figure 1E). To validate these GeneChip results, qRT‐PCR was applied to validate the levels of the top 5 lncRNAs, including NONHSAT051892, NONHSAT102457, NONHSAT141192.2, NONHSAT121332, and NONHSAT098514. The data showed an agreement in the expression levels of these genes between the GeneChip analysis, with NONHSAT141192.2 showing the most significant upregulation in U251‐GSC and LN229‐GSC cells (Figure 1F).

In addition, we isolated and cultured the GSC03 cells and differentiated GSCs (dGSCs) from patient‐derived GBM tissues (Figure 1G). Western blotting analysis demonstrated that GSC03 cells expressed higher levels of CD133, OCT4, SOX2, and Nestin than dGSCs (Figure 1H). Furthermore, the expression of NONHSAT141192.2 was remarkably increased in the patient‐derived GSC03 cells relative to dGSCs (Figure 1I). Moreover, by measuring NONHSAT141192.2 in a cohort of glioma tissues, we found that NONHSAT141192.2 was highly expressed in GBM tissues relative to I –III glioma (Figure 1J). Furthermore, Fisher's exact test showed that high expression levels of NONHSAT141192.2 were closely associated with Grade IV and Ki‐67 expression (Table 3). Additionally, FISH assays showed that NONHSAT141192.2 was expressed in both the nucleus and cytoplasm in U251‐GSC and GSC03 (Figure 1K). These results suggest that NONHSAT141192.2 is associated with the maintenance of GBM cell stemness.

**TABLE 3:** Associations between the expression level of NONHSAT141192.2 and clinicopathological parameters.

Characteristic	*n*	NONHSAT141192.2		*P* value
		High expression (*n* = 9)	Low expression (*n* = 8)	
Age				0.057
< 60	8	2 (25.0%)	6 (75.0%)	
> = 60	9	7 (77.8%)	2 (22.2%)	
Sex				0.347
Male	9	6 (66.7%)	3 (33.3%)	
Female	8	3 (37.5%)	5 (62.5%)	
Grade				**0.009**
I–III	4	0 (0.00%)	5 (100.0%)	
IV	13	9 (75.0%)	3 (25.0%)	
KPS score				1.000
< 80	9	5 (55.6%)	4 (44.4%)	
≥ 80	8	4 (50.0%)	4 (50.0%)	
Location				0.335
Left	10	4 (40.0%)	6 (60.0%)	
Right	7	5 (71.4%)	2 (28.6%)	
Size				0.637
< 3.5 cm	7	3 (42.9%)	4 (57.1%)	
≥ 3.5 cm	10	6 (60.0%)	4 (40.0%)	
Peritumoral edema				0.620
Mild	5	2 (40.0%)	3 (60.0%)	
Serious	12	7 (58.3%)	5 (41.7%)	
Ki‐67				0.057
High	9	7 (77.8%)	2 (22.2%)	
Low	8	2 (25.0%)	6 (75.0%)	

*Note:* Significance of bold value indicates statistically significant result.

### 3.2 NONHSAT141192.2 Is Required for the Maintenance of GSC Properties

**FIGURE 2:**
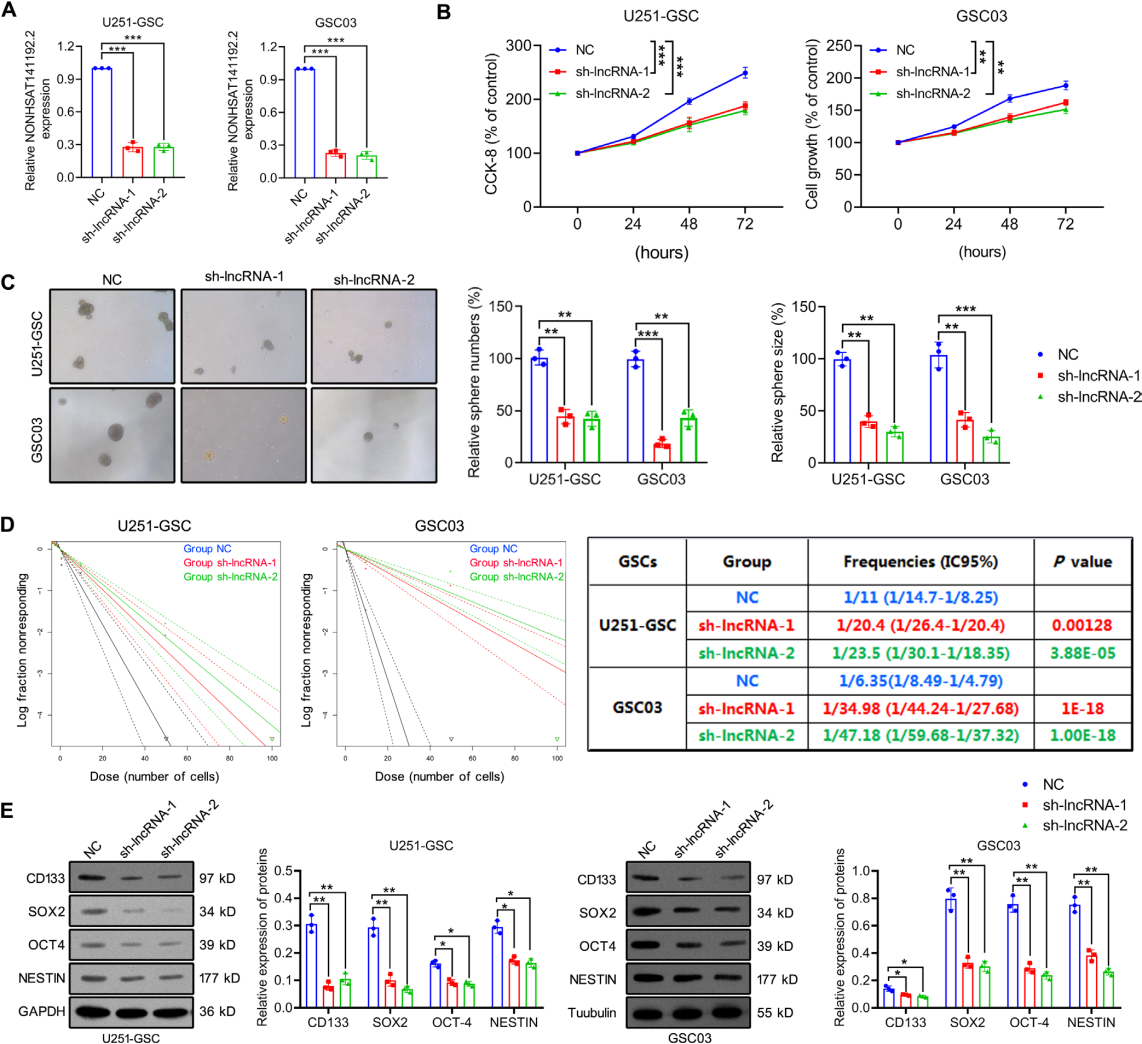
NONHSAT141192.2 knockdown inhibits the proliferation and self‐renewal of GSCs. (A) U251‐GSC and GSC03 cells were transduced with either scramble or lentivirus‐packaged NONHSAT141192.2‐specific shRNA‐1 (sh‐lncRNA‐1) or shRNA‐2 (sh‐lncRNA‐2), and NONHSAT141192.2 levels were confirmed using qRT‐PCR. (B) Cell proliferation rates of scramble or NONHSAT141192.2 shRNA‐transfected GSCs were determined using CCK‐8 assay. (C) Representative images of Tumorsphere s after 14 days of incubation. Tumorspheres were sized and analyzed using Image J. (D) Limited dilution assay with 1, 10, 20, 50, and 100 cells of NC/sh‐lncRNA‐transfected GSCs were plated in triplicates. The number of wells with no sphere was plotted as a percentage. (E) The effects of NONHSAT141192.2 knockdown on the protein levels of GSC markers were detected by Western blotting. All experiments were performed in at least three independent experiments with three replicate wells. *p < 0.05, **p < 0.01, ***p < 0.001.

To explore the role of NONHSAT141192.2 in GSCs, sh‐NONHSAT141192.2 lentivirus vectors (sh‐lncRNA‐1 and sh‐lncRNA‐2) were transfected into U251‐GSC and GSC03 cells, and the downregulated expression of NONHSAT141192.2 was validated by qRT‐PCR (Figure 2A). We analyzed the cell proliferation of GSCs using CCK‐8 assay. As shown in Figure 2B, the knockdown of NONHSAT141192.2 significantly inhibited the viability of GSCs compared to controls. Furthermore, the tumorsphere formation assay (NSFA) illustrated that NONHSAT141192.2 knockdown significantly inhibited the tumorsphere‐forming ability of U251‐GSC and GSC03 cells, which was represented by a noticeable reduction in both sphere sizes and numbers (Figure 2C). In vitro limiting dilution assay showed that NONHSAT141192.2 knockdown suppressed the GSC self‐renewal compared to negative controls (Figure 2D). Besides, Western blot analysis corroborated that NONHSAT141192.2 knockdown comparably decreased the expression of stemness markers SOX2, CD133, OCT4, and Nestin in GSCs (Figure 2E). Altogether, the preliminary studies affirmed the significant role of NONHSAT141192.2 in maintaining the malignant phenotype of GSCs.

### 3.3 Silencing NONHSAT141192.2 Radiosensitizes GSCs by Enhancing Radiation‐Induced DNA Damage

**FIGURE 3:**
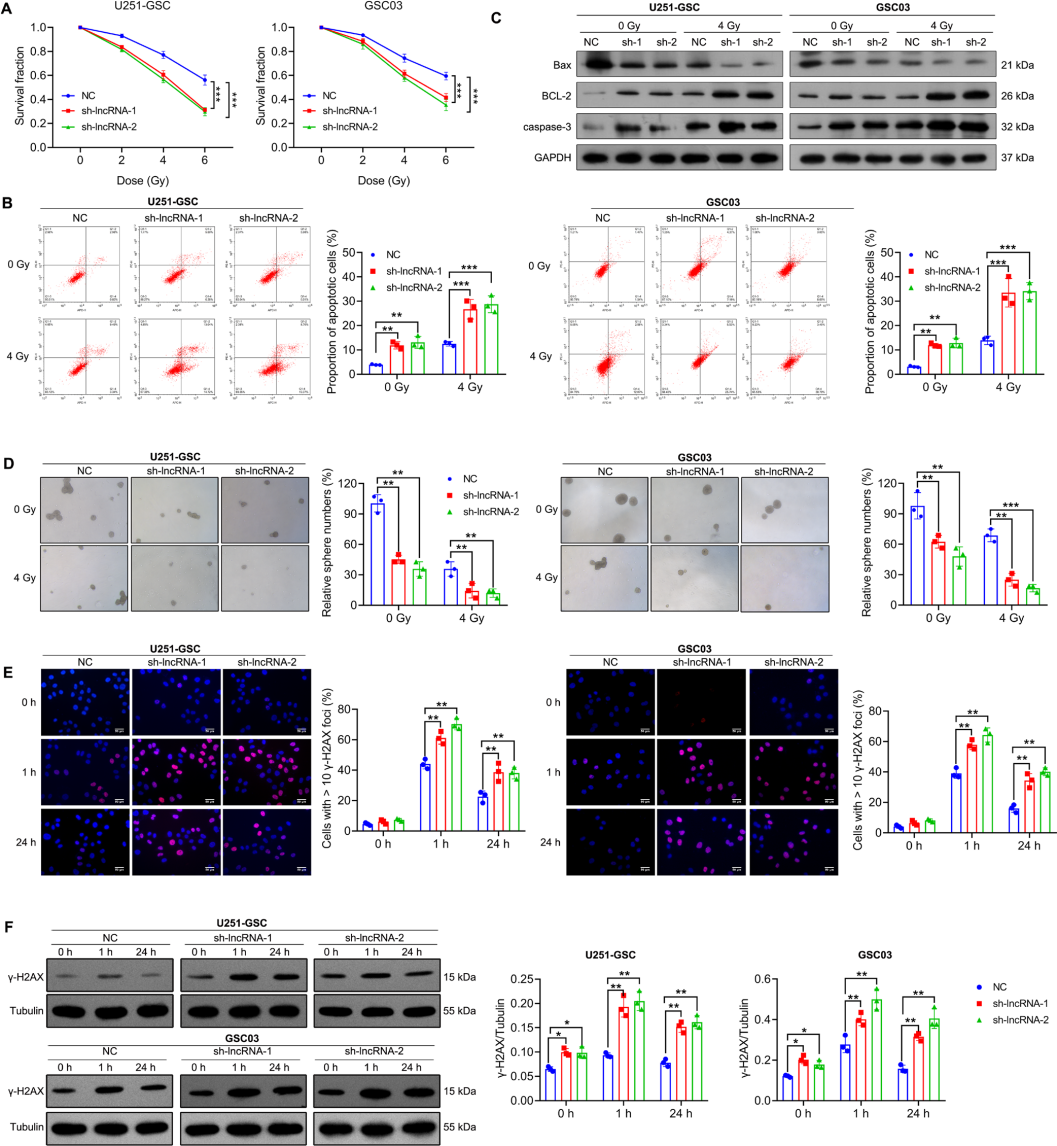
NONHSAT141192.2 knockdown enhances the radiosensitivity of GSCs. (A) CCK‐8 assay of NONHSAT141192.2‐silenced U251‐GSC and GSC03 treated with 0 2, 4, or 6 Gy IR. (B) Flow cytometry analyzed the proportion of apoptotic cells in NONHSAT141192.2‐silenced U251‐GSC and GSC03 cells post 4 Gy IR. (C) Western blotting analysis of BAX, Bcl‐2 and caspase‐3 in NONHSAT141192.2‐silenced U251‐GSC and GSC03 cells 24 h following 4‐Gy IR. (D) Tumorsphere formation assays of NONHSAT141192.2‐silenced U251‐GSC and GSC03 cells after irradiation with 0 or 4 Gy. Representative images of tumor spheres are shown. *n* = 3 wells/group. (E) Immunofluorescence staining of γ‐H2AX foci in NONHSAT141192.2‐silenced U251‐GSC and GSC03 cells at 0, 1, and 24 h post‐4‐Gy irradiation. Representative images of γ‐H2AX foci (red) and DAPI (blue) are shown. Scale bar = 50 μm. (F) Western blotting analysis of γ‐H2AX in NONHSAT141192.2‐silenced U251‐GSC and GSC03 cells 24 h following 4‐Gy IR. All experiments were performed in at least three independent experiments. *p < 0.05, **p < 0.01, ***p < 0.001.

GSCs often exhibit resistance to radiotherapy, contributing to treatment failure and the recurrence of GBM [20]. Here, we investigated the effect of NONHSAT141192.2 on the radiotherapy response of GSCs. U251‐GSC and GSC03 cells with stable knockdown of NONHSAT141192.2 (sh‐lncRNA) were irradiated with 0, 2, 4, or 6 Gy, followed by CCK‐8 experiments. As shown in Figure 3A, with increasing radiation doses, the proportion of viable cells in each group exhibited a gradual decline. Notably, under conditions of equivalent radiation dosage, the percentage of viable cells was significantly reduced in the group subjected to silencing NONHSAT14119.2.2 compared to the negative control (NC) group. Moreover, targeted silencing of NONHSAT141192.2 resulted in a considerable increase in IR‐induced apoptotic cells (Figure 3B), an elevated expression of Bcl‐2 and caspase‐3, and a reduced expression of Bax (Figure 3C), suggesting that silencing NONHSAT141192.2 enhanced the sensitivity to radiotherapy. Furthermore, the tumorsphere formation assay demonstrated that down‐regulation of NONHSAT141192.2 expression inhibited tumorsphere formation of GSCs post‐irradiation (Figure 3D).

Radiation plays an important role by inducing DNA damage, especially double‐strand breaks. Gamma histone H2AX (γH2AX) is an important marker for DNA double‐strand break repair and is widely used to evaluate the efficacy of radiotherapy. The expression and formation of γ‐H2AX reflect the cellular response to radiation damage and the process of DNA repair [20]. Therefore, to investigate the role of NONHSAT141192.2 in the regulation of DNA damage response, we performed immunofluorescence assays and Western blot analyses to assess the levels of γ‐H2AX following ionizing radiation. As shown in Figure 3E, NONHSAT141192.2‐knockdown GSCs exhibited a sustained γ‐H2AX signal compared to the NC cells after exposure to IR. Notably, at 1 h post‐IR, the γ‐H2AX signal in NONHSAT141192.2‐knockdown cells was significantly increased relative to NC cells. This elevated γ‐H2AX signaling persisted at 24 h post‐IR, while NC cells showed only a weak γ‐H2AX signal. The delayed resolution of the γ‐H2AX signal in NONHSAT141192.2‐knockdown cells indicates an accumulation of DNA damage following IR treatment. Moreover, Western blot analysis corroborated these findings, revealing a significant increase in γ‐H2AX expression in both U251‐GSC and GSC03 cells upon NONHSAT141192.2 knockdown (Figure 3F). These results collectively suggest that the knockdown of NONHSAT141192.2 enhances IR‐induced DNA damage, further implicating NONHSAT141192.2 in the modulation of cellular responses to DNA damage.

### 3.4 Targeting NONHSAT141192.2 Improves the Efficacy of Radiotherapy in an Intracranial Mouse Model

**FIGURE 4:**
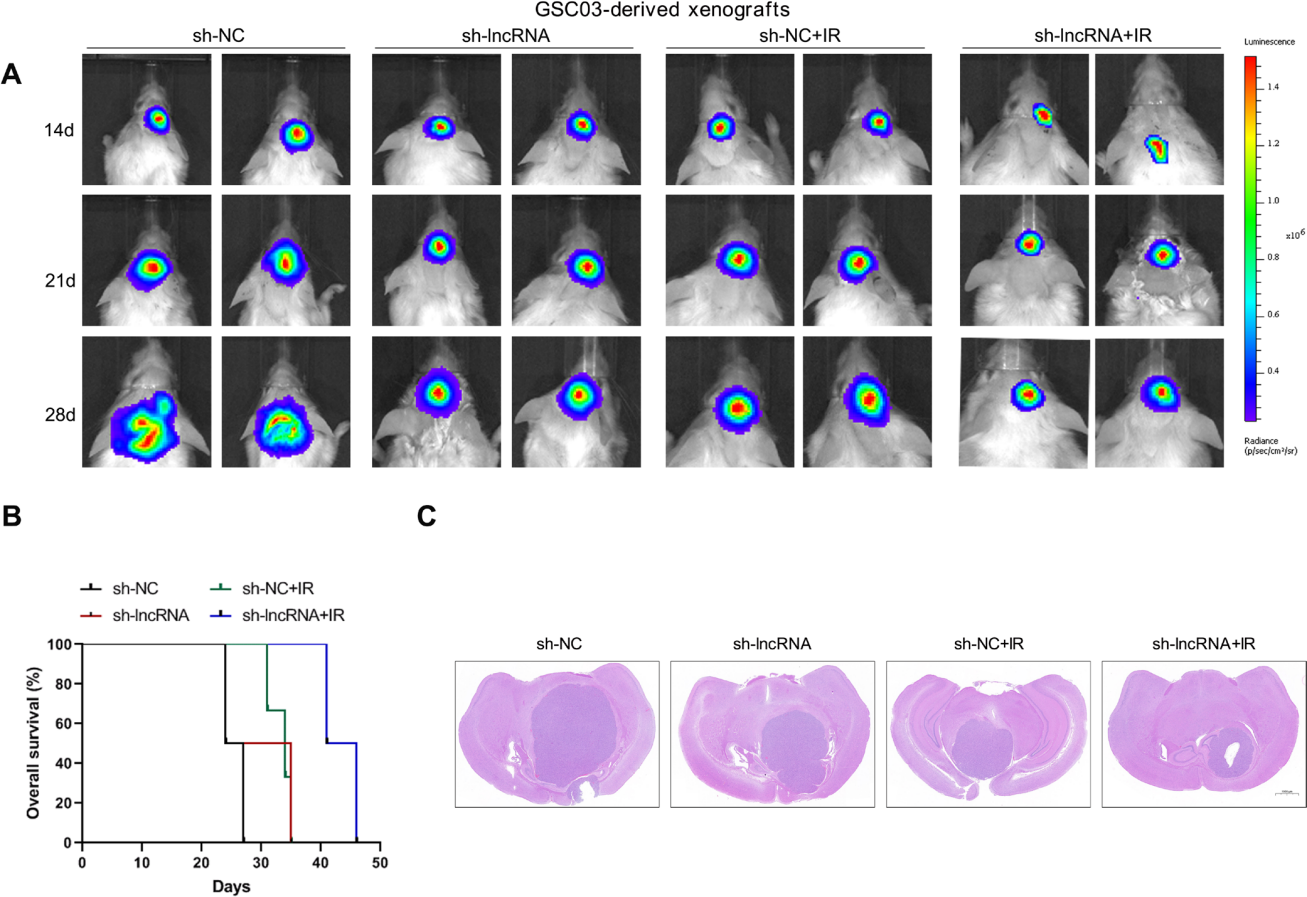
NONHSAT141192.2 knockdown reduces tumor growth and improves animal survival. (A) Representative bioluminescent imaging of intracranial tumor growth of mice bearing GBM xenografts derived from the GSC03 cells expressing sh‐NC or sh‐lncRNA shRNAs (*n =* 6 mice for each group). (B) Kaplan–Meier survival curves of mice intracranially implanted with GSC03 cells transduced with sh‐lncRNA or sh‐NC in combination with IR exposure (*n* = 6 mice for each group). Median survival: sh‐NC, 27 days; sh‐lncRNA, 33 days; sh‐NC + IR, 34 days; sh‐lncRNA + IR, 46 days. Knockdown of NONHSAT141192.2 in GSCs markedly attenuated the increased survival of mice bearing the GSC‐derived GBMs. (C) The combined effect of NONHSAT141192.2 knockdown and IR treatment in mouse xenograft tumors. Brains were harvested 20 days after transplantation. Scale bar: 1 mm. Representative data from GSC03‐derived xenografts are shown.

To investigate the function of NONHSAT141192.21 in vivo, GSC03 cells with or without NONHSAT141192.2 knockdown were implanted into the brains of SCID mice (30,000 cells per mouse). After one week, some mice were exposed to ionizing radiation (IR; 2 Gy daily for 5 days). Bioluminescent imaging revealed significant delays in the growth of the sh‐lncRNA‐expressing GSC03‐derived intracranial tumors relative to the sh‐NC tumors under either normal or IR conditions (Figure 4A). Consistently, the mice bearing the sh‐lncRNA‐expressing GSC03‐derived xenografts survived significantly longer than those in the sh‐NC group under both normal and IR conditions (Figure 4B). Moreover, NONHSAT141192.2 knockdown combined with radiation treatment further slowed tumor growth (Figure 4C). Thus, targeting NONHSAT141192.2 can increase the biological response to IR treatment.

### 3.5 NONHSAT141192.2 Sponges miR‐4279 to Downregulate Its Expression

**FIGURE 5:**
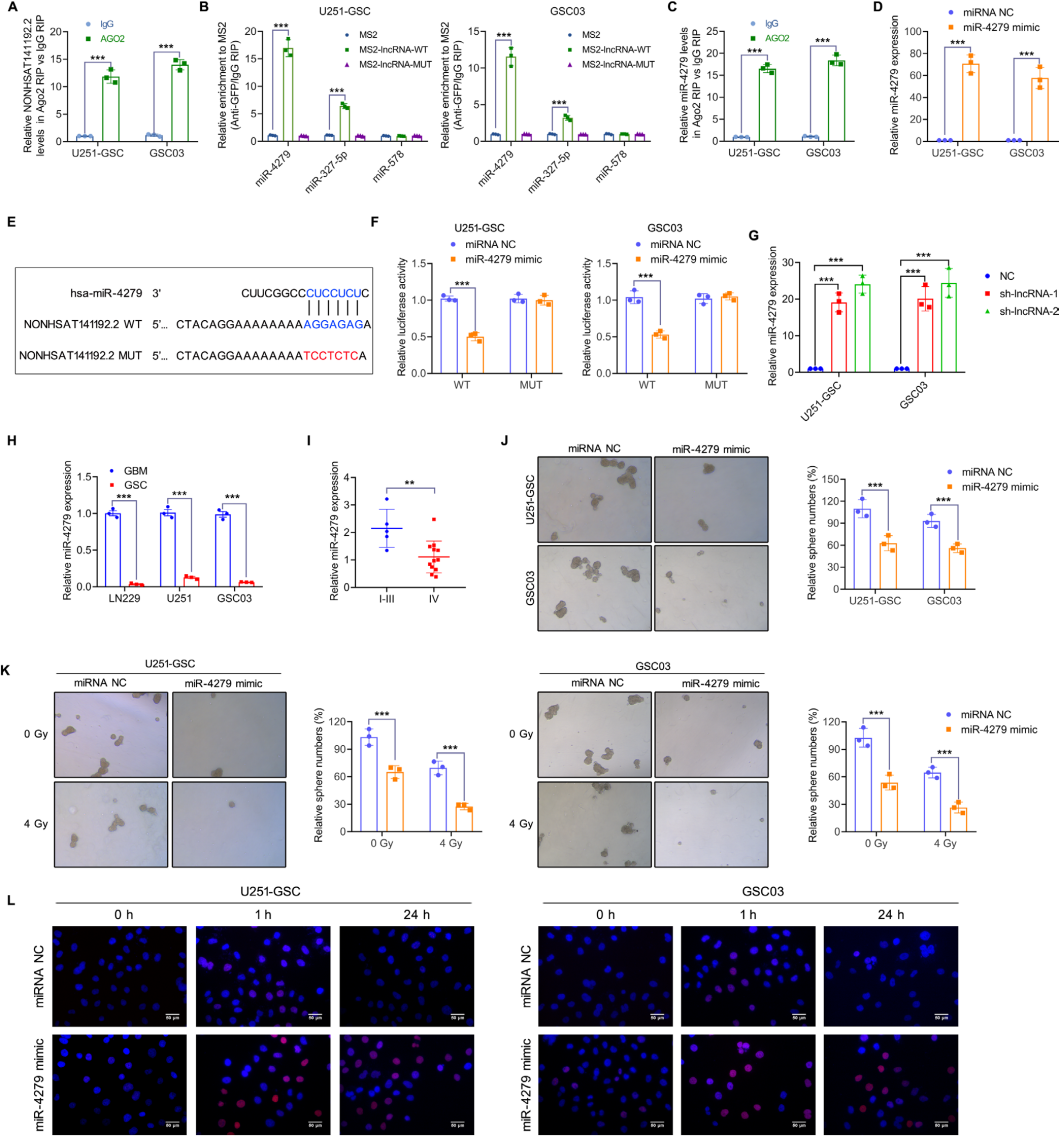
NONHSAT141192.2 sponges miR‐4279 to downregulate its expression. (A) Ago2 RIP assay demonstrates the specific enrichment of NONHSAT141192.2 in the RNA products immunoprecipitated by the anti‐AGO antibody. (B) MS2‐RIP assay demonstrated the enrichment of miR‐4279 in the RNA products captured by the NONHSAT141192.2 probe. (C) Ago2 RIP assay demonstrates the specific enrichment of miR‐4279 in the RNA products immunoprecipitated by the anti‐AGO antibody. (D) Expression of miR‐4279 in U251‐GSC and GSC03 cells transfected with miR‐4279 mimics. (E) The predicted binding sites between NONHSAT141192.2 and miR‐4279 are depicted. (F) Dual‐luciferase reporter assays were conducted to evaluate the interaction between miR‐4279 and NONHSAT141192.2 in U251‐GSC and GSC03 cells. (G) Expression levels of miR‐4279 was quantified in NONHSAT141192.2‐knockdown U251‐GSC and GSC03 cells by qRT–PCR. (H) Expression levels of miR‐4279 were quantified in both differentiated and undifferentiated U251‐GSC and GSC03 cells by qRT‐PCR. (I) The levels of miR‐4279 were assessed in I‐III glioma and GBM tissues. (J) Tumorsphere formation assays of U251‐GSC and GSC03 cells transduced with miR‐4279 mimics. Representative images of tumor spheres are shown. (K) Tumorsphere formation assays of miR‐4279‐overexpressed U251‐GSC and GSC03 cells after irradiation with 0 or 4 Gy. Representative images of tumor spheres are shown. *n* = 3 wells/group. (L) Immunofluorescence staining of γ‐H2AX foci in U251‐GSC and GSC03 cells transfected with miR‐4279 mimics at 0, 1, and 24 h post‐4‐Gy irradiation. Representative images of γ‐H2AX foci (red) and DAPI (blue) are shown. Scale bar = 50 μm. All experiments were performed in at least three independent experiments. ***p < 0.001.

Next, we explored the molecular mechanism by which NONHSAT141192.2 regulates the stemness and radioresistance of GSCs. Considering that NONHSAT141192.2 is primarily localized to the cytoplasm, we hypothesize that NONHSAT141192.2 might act as a molecular sponge to absorb miRNAs and exert biological functions. A RIP assay was performed using an anti‐Ago2 antibody, and NONHSAT141192.2 was found to be enriched in the Ago2‐pulled down RNA products (Figure 5A), suggesting that NONHSAT141192.2 can absorb miRNAs via the Ago2 complex. The downstream target miRNAs of NONHSAT141192.2 were predicted using RegRNA 2.0 (http://regrna2.mbc.nctu.edu.tw/detection.html), and miR‐4279, miR‐372‐5p, and miR‐578 were predicted to be the downstream targets of NONHSAT141192.2. The MS2‐RIP assay showed miR‐4279 had a high binding efficiency and affinity for NONHSAT141192.2 (Figure 5B). Furthermore, miR‐4279 was enriched in the Ago2‐pulled down RNA products (Figure 5C), indicating a specific interaction between miR‐4279 and NONHSAT141192.2. To identify the specific binding region between NONHSAT141192.2 and miR‐4279, miR‐4279 was overexpressed by transfecting the miR‐4279 mimic into U251‐GSC and GSC03 cells (Figure 5D). A dual‐luciferase reporter system was constructed by inserting the wild type (WT) and mutant (MUT) sequence of NONHSAT141192.2 into the luciferase plasmids (Figure 5E) and miR‐4279 was overexpressed following transfection with the miRNA mimic. Results of the dual‐luciferase assay indicated that the miR‐4279 mimic remarkably reduced the luciferase activity of NONHSAT141192.2‐Wt in U251‐GSC and GSC03 cells; However, no significant differences in the luciferase activity of NONHSAT141192.2‐Mut were observed following miR‐4279 overexpression (Figure 5F). In addition, miR‐4279 expression was upregulated in the sh‐lncRNA group, whereas not in the sh‐NC group (Figure 5G). A lower expression level of miR‐4279 was observed in the GSCs compared to their differentiated state (Figure 5H). By measuring miR‐4279 expression in a cohort of glioma tissues, we found that the expression of miR‐4279 was decreased in GBM tissues compared with I –III glioma tissues (Figure 5I).

Then, we investigated the role of miR‐4279 in the stem cell properties and radioresistance of GSCs. The tumorsphere formation assay showed that the miR‐4279 mimic inhibited the tumorsphere‐forming ability of GSCs (Figure 5J). Further experiments demonstrated that miR‐4279 overexpression enhanced radiosensitivity, which was represented by a significant decrease in tumorsphere numbers (Figure 5K). Furthermore, IF staining showed that, after 24 h of IR treatment, the number of γ‐H2AX foci in cells with overexpressed miR‐4279 was significantly increased compared to the control group (Figure 5L). Taken together, these findings suggest that miR‐4279 inhibits the stem cell properties and radioresistance of GSCs.

### 3.6 Transcriptomics Analysis Reveals That NONHSAT141192.2/miR‐4279 Axis Regulates the Expression of SOX2 and PIK3R3

**FIGURE 6:**
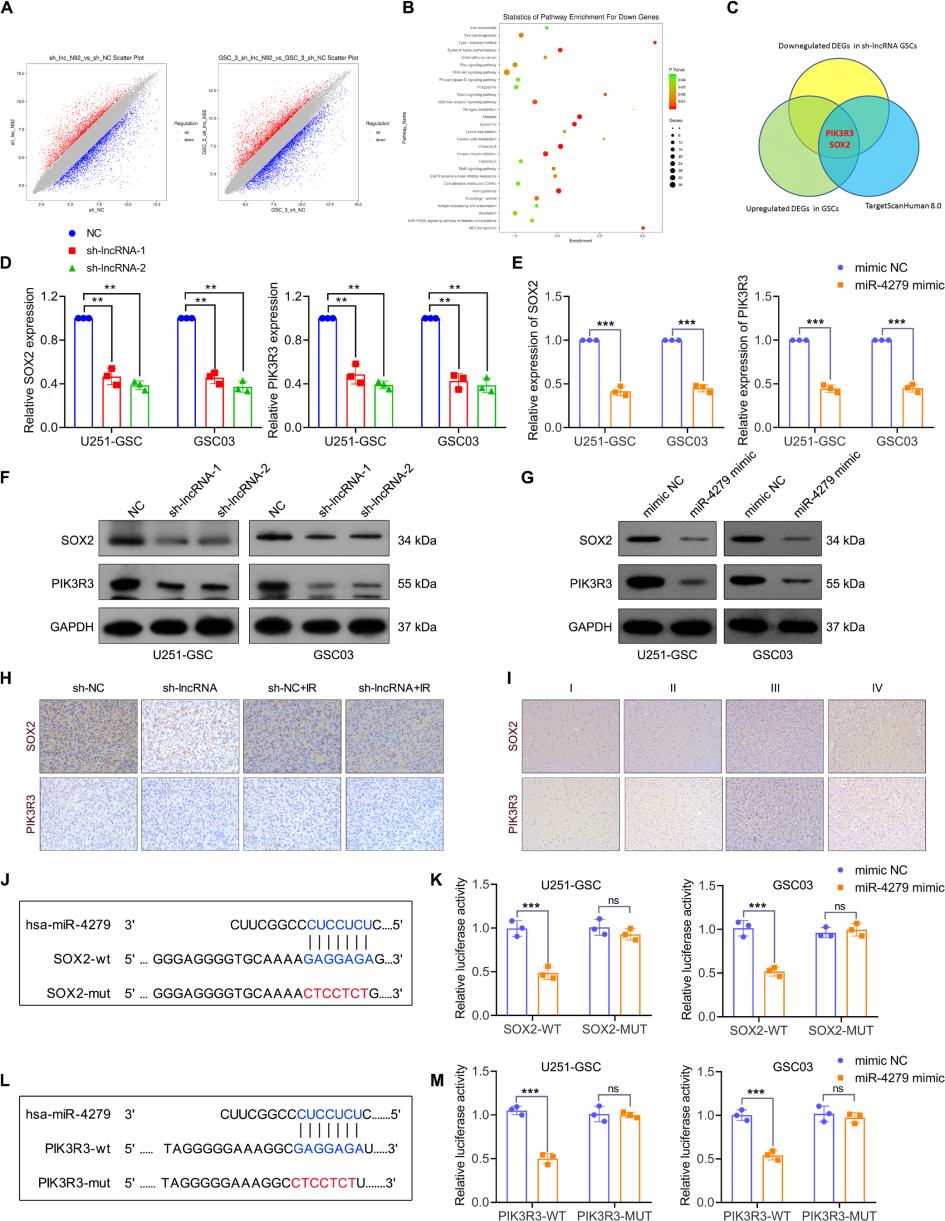
SOX2 and PIK3R3 are identified as the potential targets of NONHSAT141192.2/miR‐4279 axis. (A) Scatter plots of differentially expressed genes (DEGs) between NC and (B) KEGG pathways enrichment for downregulated DEGs in NONHSAT141192.2‐knockdown GSCs. (C) The Venn diagram shows that PIK3R3 and SOX2 are potential target genes of miR‐4279 that are highly expressed in GSCs and decrease in NONHSAT141192.2‐knockdown GSCs. (D) QRT‐PCR analysis of SOX2 and PIK3R3 mRNAs in NONHSAT141192.2‐knockdown GSC cells (*n* = 3). (E) QRT‐PCR analysis of SOX2 and PIK3R3 mRNAs in GSC cells with miR‐4279 overexpression (*n* = 3). (F) Western Blot analysis of SOX2 and PIK3R3 proteins in NONHSAT141192.2‐knockdown GSC cells (*n* = 3). (G) Western Blot analysis of SOX2 and PIK3R3 proteins in GSC cells with miR‐4279 overexpression (*n* = 3). (H) IHC staining of SOX2 and PIK3R3 in sh‐NC and sh‐lncRNA mouse xenografts (*n* = 3). (I) IHC assay of 17 tumor tissues of patients with glioma. (J) The predicted binding sites between SOX2 and miR‐4279 are depicted. (K) Dual‐luciferase reporter assays were conducted to evaluate the interaction between miR‐4279 and SOX2 in U251‐GSC and GSC03 cells (*n* = 3). (L) The predicted binding sites between PIK3R3 and miR‐4279 are depicted. (M) Dual‐luciferase reporter assays were conducted to evaluate the interaction between miR‐4279 and PIK3R3 in U251‐GSC and GSC03 cells (*n* = 3). **p < 0.01, ***p < 0.001. ns, no significance.

To explore the specific mechanism by which NONHSAT141192.2 maintains GSC properties and radioresistance, transcriptome sequencing analysis was performed on NONHSAT141192.2‐silenced U251‐GSC and GSC03 cells and relative control cells. Overall, 3767 differentially expressed genes (DEGs) were identified in both cell lines, with 1724 upregulated DEGs and 2043 downregulated DEGs in the NONHSAT141192.2‐KD group when compared with control cells (Adj *p* < 0.05; |log2fold change| > 2) (Figure 6A). KEGG enrichment analysis showed that downregulated DEGs in the NONHSAT141192.2‐KD group were mainly enriched in the Ras, PI3K‐Akt, phospholipase D, Notch, and NOD‐like receptor signaling pathways (Figure 6B). Among the 547 upregulated DEGs in GSCs and 2043 downregulated DEGs in NONHSAT141192.2‐KD GSCs 281 genes were identified. To identify potential target genes of miR‐4279, we utilized the TargetScanHuman 8.0 (https://www.targetscan.org/vert_80/) database to predict miRNA‐targeted mRNAs. This approach allowed us to narrow down the list of candidate mRNAs by intersecting the predicted targets with genes that were found to be upregulated in GSC cells according to previous GeneChip data and simultaneously downregulated in the NKN knockdown group. Through this intersection analysis, SOX2 and PIK3R3 were identified as key genes of interest (Figure 6C). QRT‐PCR analysis verified that the expression of SOX2 and PIK3R3 was reduced when U251‐GSCs or GSC03 cells were transfected with sh‐lncRNA or miR‐4279 (Figure 6D,E). Western blot analysis showed the reduced protein expression of SOX2 and PIK3R3 when U251‐GSCs or GSC03 cells were transfected with sh‐lncRNA or miR‐4279 (Figure 6F,G). Additionally, consistent with our in vitro findings, tumors from NONHSAT141192.2‐KD mice showed decreased levels of SOX2 and PIK3R3 staining (Figure 6H). Furthermore, the immunohistochemistry analysis of 17 glioma specimens revealed a notably higher incidence of positive expression for SOX2 and PIK3R3 in GBM tissues compared to I‐III glioma tissues (Figure 6I).

To verify the binding relationship between SOX2 and miR‐4279, dual‐luciferase reporter plasmids were constructed by inserting the wild type (wt) and mutant (mut) sequence of the 3′‐UTR of SOX2 into the luciferase plasmids (Figure 6J). Results of the dual‐luciferase assay indicated that the miR‐4279 mimic remarkably reduced the luciferase activity of SOX2‐wt in U251‐GSC and GSC03 cells; However, no significant differences in the luciferase activity of SOX2‐mut were observed following miR‐4279 overexpression (Figure 6K). The luciferase plasmids containing PIK3R3‐wt and PIK3R3‐mut sequences were also constructed as shown in Figure 6L, and the dual‐luciferase assay confirmed that the miR‐4279 mimic remarkably reduced the luciferase activity of PIK3R3‐wt in U251‐GSC and GSC03 cells but had no effect on the luciferase activity of PIK3R3‐mut plasmids (Figure 6M). These results demonstrate that miR‐4279 physically interacts with SOX2 and PIK3R3 in GSC cells.

### 3.7 The Role of NONHSAT141192.2‐miR‐4279‐PIK3R3/SOX2 Axis in Radioresistance of GSGs

**FIGURE 7:**
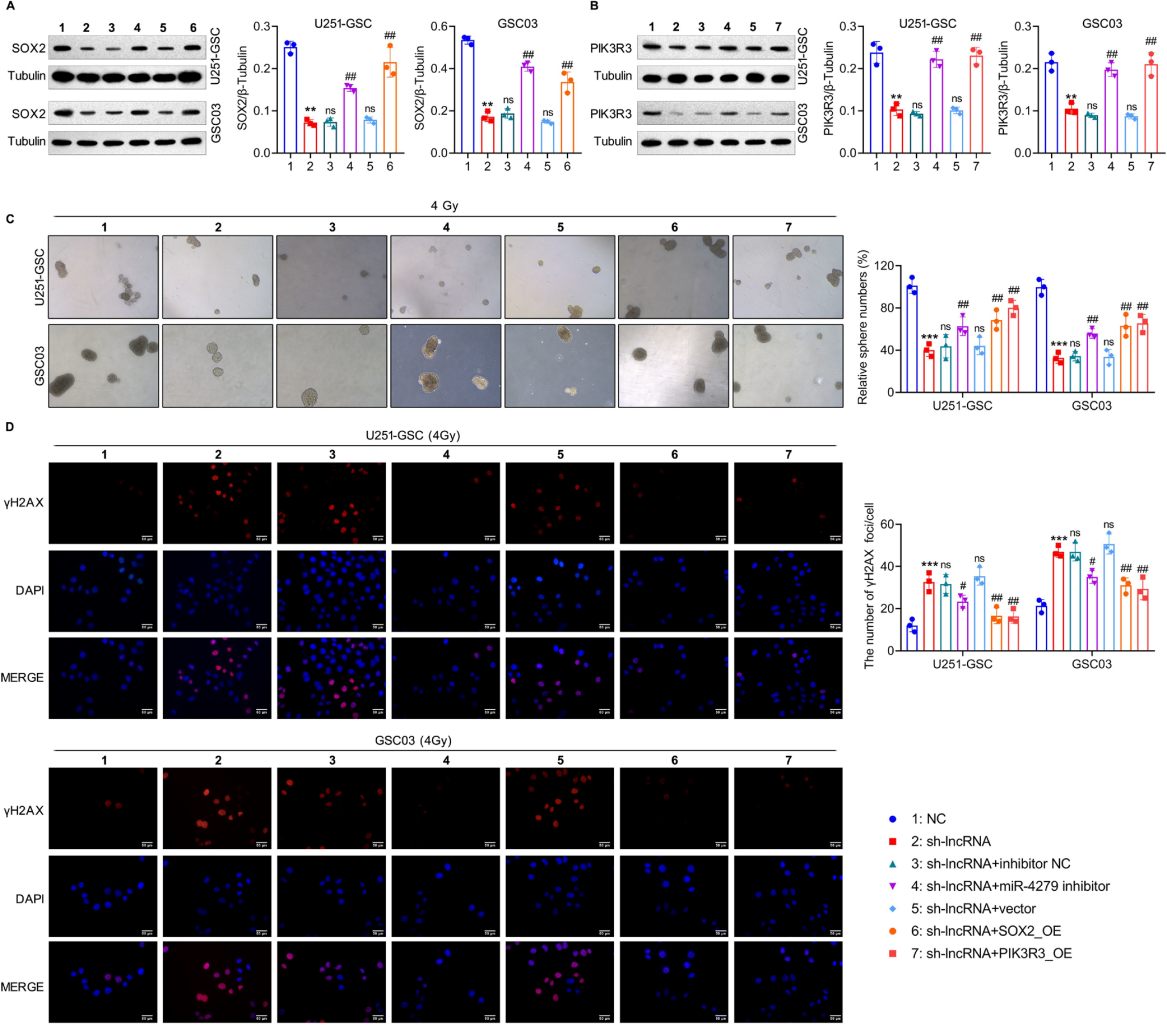
The role of NONHSAT141192.2‐miR‐4279‐PIK3R3/SOX2 axis in the stemness and radioresistance in GSCs. (A) Western blot analysis of SOX2 in U251‐GSC and GSC03 cells co‐transduced with NONHSAT141192.2 shRNA and miR‐4279 inhibitor, SOX2‐overexpressing plasmids (SOX2_OE). (B) Western blot analysis of SOX2 in U251‐GSC and GSC03 cells co‐transduced with NONHSAT141192.2 shRNA and miR‐4279 inhibitor, PIK3R3‐overexpressing plasmids (PIK3R3_OE). (C) Tumorsphere formation assays of NONHSAT141192.2‐knockdown U251‐GSC and GSC03 cells co‐transduced with miR‐4279 inhibitor, SOX2_OE or PIK3R3_OE. Representative images of tumor spheres are shown. (D) Immunofluorescence staining of γ‐H2AX foci in NONHSAT141192.2‐knockdown U251‐GSC and GSC03 cells co‐transduced with miR‐4279 inhibitor, SOX2_OE or PIK3R3_OE at 24 h post‐4‐Gy irradiation. Representative images of γ‐H2AX foci (red) and DAPI (blue) are shown. Scale bar = 50 μm. All experiments were performed in at least three independent experiments. **p < 0.01, ***p < 0.001. vs. the NC groups. ns, no significant, ##*p* < 0.01, vs. the sh‐lncRNA groups.

To verify whether miR‐4279, PIK3R3, or SOX2 was essential for NONHSAT141192.2‐mediated radioresistance, rescue experiments were transfected with miR‐4279 inhibitor, PIK3R3_OE, or SOX2_OE in NONHSAT141192.2‐silencing GSCs. Western blot analysis indicated that miR‐4279 inhibitor, SOX2_OE, or PIK3R3_OE promoted the expression of SOX2 or PIK3R3 in NONHSAT141192.2‐silencing GSCs (Figure 7A,B). Clonogenic assays showed that NONHSAT141192.2‐silencing‐mediated radiosensitivity was largely abrogated by miR‐4279 inhibitor, SOX2_ overexpression, or PIK3R3 overexpression (Figure 7C). Concerning radiosensitivity, GSCs with NONHSAT141192.2 silencing had significantly increased γ‐H2AX foci‐forming ability post‐IR, whereas miR‐4279 inhibitor, SOX2_OE, or PIK3R3_OE largely abrogated NONHSAT141192.2‐silencing‐induced DNA damage in GSCs (Figure 7D). In summary, these results indicated that NONHSAT141192.2 inhibits DNA damage and promotes radioresistance of GSCs via regulating SOX2 or PIK3R3 expressions.

## 4 Discussion

GBM is known for its aggressive nature and dismal prognosis, largely due to the presence of GSCs, which contribute significantly to tumor initiation, recurrence, metastasis, and resistance to conventional therapies [21, 22, 23]. While recent advances have illuminated the role of lncRNAs in regulating various aspects of GBM biology, the specific lncRNAs associated with GSCs remained largely unexplored. In this study, we identify NONHSAT141192.2 as a novel, dysregulated lncRNA in GSCs and elucidate its critical role in promoting GBM progression, stemness, and radioresistance. Our findings provide insights into potential therapeutic strategies, based on the highlighted role of NONHSAT141192.2 in GSC biology.

Firstly, we observed that the expression of NONHSAT141192.2 was significantly higher compared to other lncRNAs in GSCs through GeneChip analysis screening and qRT‐PCR validation. This suggests a potential association between NONHSAT141192.2 and the stem cell properties characteristic of GSCs. Moreover, its elevated expression was also found in aggressive GBM tissues relative to lower‐grade gliomas (grades I–III), and high expression of NONHSAT141192.2 correlated with Ki‐67 positive expression, indicating its potential involvement in more malignant forms of the disease. These observations prompted us to further investigate the functional roles of this lncRNA.

To investigate its function, we knocked down NONHSAT141192.2 in U251‐GSCs, which were derived from U251 cells and primary GSC03 cells from patients. Silencing NONHSAT141192.2 significantly reduced cell proliferation, tumorsphere formation, and self‐renewal capacity, which are all key characteristics of GSCs. This suggests that targeting this lncRNA may effectively diminish the tumor‐initiating potential of GBM. Our results align with previous research that underscores the role of long non‐coding RNAs in regulating stemness. For example, lncRNA LINC01503 enhances the cancer stem cell properties of GBM cells [24]. Similarly, LncRNA NEAT1 has also been implicated in promoting GSC malignancy [25]. Furthermore, NONHSAT141192.2 knockdown also led to a marked decline in the expressions of key stem cell markers (such as SOX2, CD133, OCT4, and Nestin) in GSCs, reinforcing the idea that NONHSAT141192.2 plays a central role in regulating GSC stemness. This is consistent with previous reports that highlight the role of lncRNAs in regulating stemness through modulation of transcription factors like SOX2. SOX2 and OCT4 are known to be master regulators of stem cell self‐renewal, and their expressions are tightly controlled in GSCs [26, 27]. Our findings further support the notion that NONHSAT141192.2 may act as a critical mediator in the regulation of core pluripotency master regulators, thereby promoting the stem‐like phenotype of GSCs.

In addition to its impact on GSC proliferation and self‐renewal, the depletion of NONHSAT141192.2 also rendered GSCs more sensitive to radiation therapy—a critical challenge in GBM treatment. Following the knockdown of NONHSAT141192.2, we noted decreased GSC viability, impaired tumorsphere formation, and increased apoptosis when exposed to radiation in vitro. These results indicate that NONHSAT141192.2 not only supports GSC stemness but also protects these cells from the cytotoxic effects of radiation. This is particularly relevant given existing literature that highlights the enhanced DNA repair capabilities of GSCs and their resistance to radiation‐induced cell death [5, 28, 29]. Additionally, the knockdown of NONHSAT141192.2, in combination with radiation, inhibited tumor growth in intracranial xenograft models, further emphasizing its potential as a therapeutic target for increasing the effectiveness of GBM treatments amidst radioresistance.

Mechanistically, we identified a novel regulatory axis involving NONHSAT141192.2, miR‐4279, and crucial genes related to stemness and radioresistance in GSCs. By acting as a molecular sponge, NONHSAT141192.2 regulates miR‐4279 expression, which in turn influences the stem cell properties and radioresistance of GSCs through SOX2 and PIK3R3. This finding is consistent with prior studies indicating that lncRNAs can modulate gene expression by serving as decoys for miRNAs, affecting the stability and translation of target mRNAs [10, 30, 31]. Our RNA immunoprecipitation (RIP) and MS2‐RIP assays showed that NONHSAT141192.2 interacts with Ago2, a core component of the RNA‐induced silencing complex, effectively sponging miR‐4279. The specificity of this interaction was further validated through luciferase reporter assays. Importantly, the downregulation of miR‐4279 in GSCs and GBM tissues suggests that the NONHSAT141192.2/miR‐4279 axis may be disrupted in aggressive glioma subtypes, positioning NONHSAT141192.2 as a critical post‐transcriptional regulator essential for maintaining GSC stemness and radioresistance.

Through transcriptomic analysis, we also found that the NONHSAT141192.2/miR‐4279 axis regulates key genes such as SOX2 and PIK3R3, both of which are vital for GSC maintenance and radioresistance. SOX2, a well‐known marker of stemness [32, 33], was significantly downregulated in GSCs following either NONHSAT141192.2 silencing or miR‐4279 overexpression. This is consistent with literature linking high SOX2 levels to advanced tumor grades and enhanced self‐renewal and tumorigenicity in GSCs [34], [35]. Similarly, PIK3R3, a subunit of the PI3K complex involved in cell survival and proliferation, showed reduced expression following knockdown of NONHSAT141192.2 or overexpression of miR‐4279. Our dual‐luciferase reporter assays confirmed that miR‐4279 directly targets both SOX2 and PIK3R3, indicating their roles in the regulation of GSC properties and resistance to radiation. To validate the functional significance of this regulatory axis, we conducted rescue experiments in NONHSAT141192.2‐silenced GSCs. Inhibition of miR‐4279 or overexpression of SOX2 or PIK3R3 largely mitigated the increased radiosensitivity observed with NONHSAT141192.2 knockdown. These results underscore the critical function of the NONHSAT141192.2/miR‐4279/PIK3R3/SOX2 axis in managing both stemness and radioresistance in GSCs, highlighting NONHSAT141192.2's role as a key regulatory hub in GSC biology.

In addition, several key issues warrant further investigation. Firstly, while we found that the expression level of NONHSAT141192.2 was significantly higher in aggressive GBM tissues compared to low‐grade glioma tissues, we also observed variable expression patterns of NONHSAT141192.2 across different GBM samples. This variability may reflect the inherent heterogeneity of GBM or differences in the tumor microenvironment, which can influence lncRNA expression. Future studies involving larger patient cohorts will be essential to clarify the specific factors driving this variability and to assess the clinical significance of NONHSAT141192.2 as a potential biomarker for GBM. Furthermore, it is important to note that our study focused exclusively on radiation resistance rather than chemoresistance. Our preliminary findings indicated a more pronounced effect of NONHSAT141192.2 on radiation sensitivity in our model system. However, both forms of therapy resistance present critical challenges in the treatment of GBM. Therefore, future research should explore the role of NONHSAT141192.2 in chemoresistance to provide a more holistic view of treatment response in GBM. Lastly, conducting a more comprehensive examination of the miRNA‐lncRNA‐protein network will significantly enhance our molecular understanding of NONHSAT141192.2's regulatory mechanisms. To this end, we plan to continue characterizing these networks through bioinformatics analyses and experimental validation in future studies. This approach will deepen our insights into how NONHSAT141192.2 contributes to glioma stem cell biology and the mechanisms underlying resistance to therapies.

## 5 Conclusion

In summary, we demonstrate that NONHSAT141192.2 is upregulated in GSCs and aggressive GBM tissues, and its silencing leads to reduced GSC proliferation, stemness maintenance, and radioresistance. Additionally, depletion of NONHSAT141192.2 inhibits tumor growth and enhances radiosensitivity in xenograft models. Mechanistically, NONHSAT141192.2 promotes the expression of SOX2 and PIK3R3 via sponging miR‐4279, thereby linking it to stemness and radioresistance. These findings suggest NONHSAT141192.2 may serve as a potential therapeutic target for GBM treatment and radiosensitization strategies.

## Author Contributions

Sihan Wang, Haolang Ming, Zhen Wang, and Xinyue Zhang. wrote the main manuscript text. Sihan Wang and Haolang Ming prepared Figures 1 and 2. Zengguang Wang, Xinyue Zhang, Di Wu, and Yin Bo prepared Figures 3 and 4. Hang Wang and Yuanbo Luo prepared Figure 5. Zhenfeng Han, Lingyu Hao, and Yijia Xiang. prepared Figure 6. Xu Han, Zengguang Wang, and Yi Wang prepared Figure 7.

## Ethics Statement

For patients: All procedures involving human subjects were conducted following the Helsinki Declaration as revised in 2013 and with the approval of the ethics committee of Tianjin Medical University General Hospital (Approval No: IRB2022‐KY‐234; Date: 20220310). For animals: All procedures involving animals complied with the statement for Use of Animals in the Basel Declaration, and ethical approval was granted by the ethics committee of Tianjin Medical University General Hospital (Approval No: IRB2022‐DWFL‐329; Date: 20220310).

## Consent

All authors agree with the publication of the manuscript. Written informed consent was obtained from all participants.

## Conflicts of Interest

The authors declare no Conflicts of Interest.

## Data Availability

The authors have nothing to report.
